# Cutting Out the Gaps Between Proteases and Programmed Cell Death

**DOI:** 10.3389/fpls.2019.00704

**Published:** 2019-06-04

**Authors:** Anastasia V. Balakireva, Andrey A. Zamyatnin

**Affiliations:** ^1^Institute of Molecular Medicine, I.M. Sechenov First Moscow State Medical University, Moscow, Russia; ^2^Belozersky Institute of Physico-Chemical Biology, Lomonosov Moscow State University, Moscow, Russia

**Keywords:** protease, cascade, processing, caspase, programmed cell death, plant, metacaspase

## Abstract

To date, many animal models for programmed cell death (PCD) have been extensively characterized and classified while such efforts in plant types of PCD still remain poorly understood. However, despite a wide range of functional differences between PCD types in animals and plants, it is certain that all of them are regulated through the recruitment of proteases. Most importantly, proteases are able to perform proteolysis that results in a gain or loss of protein function. This principle relies on the presence of proteolytic cascades where proteases are activated upon various upstream stimuli and which lead to repetitive cell death. While protease activation, proteolytic cascades and targeted substrates are described in detail mainly for nematode, human, and mice models of apoptosis, for plants, only fragmentary knowledge of protease involvement in PCD exists. However, recently, data on the regulation of general plant PCD and protease involvement have emerged which deepens our understanding of the molecular mechanisms responsible for PCD in plants. With this in mind, this article highlights major aspects of protease involvement in the execution of PCD in both animals and plants, addresses obstacles and advances in the field and proposes recommendations for further research of plant PCD.

## Introduction

Programmed cell death (PCD) is an integral part of any organism’s life, and for animals PCD has been classified into apoptosis, autophagy and necrosis (Galluzzi et al., 2018). However, when it comes to classification of plant PCD, it is a rather complex matter. Morphologically, plant forms of PCD were classified into autolytic and non-autolytic types (Van Doorn et al., 2011), and where autolytic death implies a rupture of the tonoplast with the subsequent rapid clearance of the cytoplasm that causes the death of the cell. Non-autolytic PCD is characterized by such events happening after cells gave already died (Van Doorn, 2011). Functionally, PCD may occur during the normal development of a plant (dPCD) (Van Durme and Nowack, 2016), or be triggered by pathogens (pPCD) (Huysmans et al., 2017), and which may result in a plant-specific form of PCD, for example, dying a hypersensitive response (HR) death (Balakireva and Zamyatnin, 2018).

Moreover, both dPCD and pPCD may exhibit mixed traits of autophagic, autolytic and non-autolytic forms simultaneously, which makes it difficult to distinguish dPCD and pPCD morphologically. Despite that obstacle, it is clear that the regulation of any type of PCD is held by proteases (Zamyatnin, 2015), and evidenced in both animals and plants, with apoptosis in animals being orchestrated mainly by the well-known caspases (aspartate-specific cysteine proteases). However, due to the presence of semirigid cell wall in plants, it is consequently accepted, that apoptosis is morphologically absent in plants (Locato and De Gara, 2018). Moreover, the caspases are absent in plants (Uren et al., 2000). Nevertheless, during plant PCD, caspase-like activity can be detected and is attributed to the alternative families of proteases, which include the metacaspases (Coll et al., 2014), vacuolar processing enzymes (VPEs) (Hatsugai et al., 2004, 2015), and the papain-like cysteine proteases (PLCP), etc., (Gilroy et al., 2007; Paireder et al., 2016), summarized in Supplementary Table. However, exactly how these proteases orchestrate PCD in plants is still largely unknown. In this article, we compare major aspects of protease function in PCD between animals and plants, address obstacles and advances in the field, and explore niches for research in the future.

## Protease Initiation Events: How Are PCD Proteases Activated in Plants?

**FIGURE 1:**
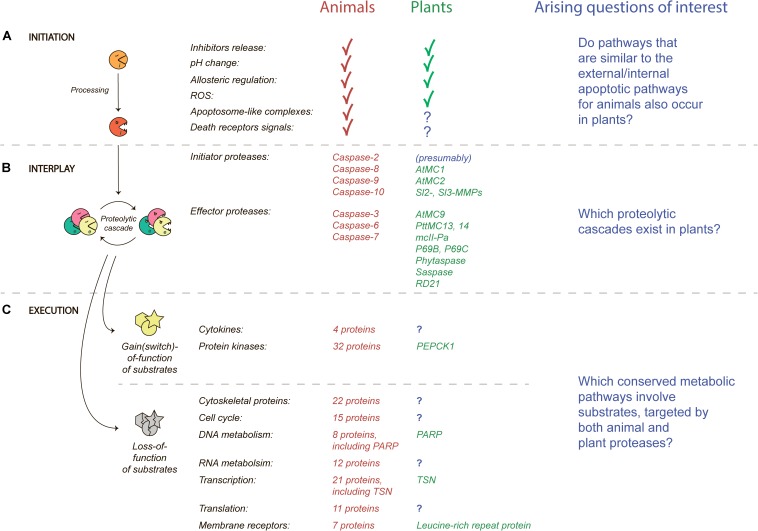
A protease implementation in PCD. **(A)** Factors that affect activation of proteases (initiation); **(B)** protease cooperation after initiation (proteolytic cascade); and **(C)** effects on signal transduction by activated proteases (execution). Groups of caspase substrates were adapted from Fischer et al. (2003). Protease names, pathways that correspond to animals are indicated in red, and for plants – in green. Arising questions of interest are indicated by question marks and text in blue.

The first step in the plant PCD by proteases is their activation, since all known PCD-related proteases in both animals and plants are synthetized as inactive zymogens and which require proteolytic processing in order to become enzymatically active. Most secreted zymogens have similar domain structures and contain a signal peptide, N-terminal and/or C-terminal autoinhibitory prodomains, and a catalytic domain. Hydrolysis of the autoinhibitory domains may happen autocatalytically or by other proteases, which triggers a conformational change that is indispensable for protease activity (Figure 1A).

### Mechanistic Similarities Between Animal and Plant Protease Activation

It is widely accepted that inhibitors are essential for the regulation of protease activity in animals (Armstrong, 2006) and is evidenced for plants too. For example, during the embryonic development of *Nicotiana tabacum*, the mechanism triggering PCD of a structure involved solely in positioning the embryo proper within the seed – suspensor – is based on the antagonistic actions of two proteins, the protease inhibitor (cystatin NtCYS), and its target (cathepsin H-like protease NtCP14) (Zhao et al., 2013). Another example is the protease “Responsive to Desiccation-21” (RD21) and the activity of which is regulated through the irreversible inhibition by AtSerpin1 during PCD (Lampl et al., 2013). Moreover, recently it has been shown that there is cross-talk between the pathways for irreversible inhibition of RD21 (by AtSerpin1) and reversible inhibition, mediated by the water-soluble chlorophyll-binding protein (WSCP), which broadly contributes to the regulatory role of RD21 in innate immunity and development (Rustgi et al., 2017).

Inhibitors and activators of certain proteases are usually co-located within the same subcellular compartments (Van der Hoorn, 2004), which can differ in pH values. This can significantly affect protease activation status. One good example illustrating this point are lysosomal proteases, known as the human cathepsins, which are activated in the low pH, acidic environments within the lysosome. Interestingly, this is evidenced for plant vacuolar proteases as well, such as the *Arabidopsis* RD21 protease (Yamada et al., 2001), or its wheat homolog triticain-α (Savvateeva et al., 2015). The cysteine C13 protease legumain, which displays low-pH-dependent dimerization and activation is also another good example (Zauner et al., 2018).

Conversely, the activity of some proteases with a neutral pH optimum does depend greatly on their calcium-binding ability as in the case of mammalian membrane-bound proteases (Mellgren, 1987), which has been evidenced for plant proteases as well. Here, phytocalpain DEK1 is a calcium-dependent membrane-bound protease, the activity of which enhances significantly after binding to calcium (van der Hoorn, 2008) and serves as a good paradigm, as do the type II metacaspases (Bozhkov et al., 2005) and the MCA2 protein from *Arabidopsis thaliana* (Watanabe and Lam, 2011).

Other activators of caspase-3 in animals are reactive oxygen species (ROS) (Higuchi et al., 1998). Similarly, ROS are able to activate proteases in plants too. For example, caspase-like proteases (C1LP and C3LP) had increased activity resulting from reactive carbonyl species (RCS) which are downstream products of ROS and which consequently triggered PCD in *N. tabacum* (Biswas and Mano, 2016). Vacuolar cell death can also be regulated by ROS as oxygen radical directly cause vacuole membrane permeabilization and the release of RD21 and its consequent binding to AtSerpin1 in *A. thaliana* cells leading to PCD (Koh et al., 2016).

Taken together, we can conclude that the activation of proteases in animals and plants can happen through very similar mechanisms, as seen in animals and based on this proposition, does raise questions about how protease initiation may be triggered in plants mechanistically.

### Does Plant Protease Activation Occur in a Similar Manner to Animals, During Cell Death?

During apoptosis, the extrinsic pathway of caspase activation requires the engagement of cell membrane receptors by a ligand, leading to the formation of the death-inducing signaling complex (DISC). The DISC activates caspase-8, which subsequently activates caspase-3 and caspase-7 (Crawford and Wells, 2011). However, it is still unknown whether such death receptors can transduce such signals directly to the proteases in plants and therefore does require further investigation.

Alternatively, the intrinsic pathway of caspase activation requires the release of mitochondrial cytochrome *c* which induces the formation of a multiprotein complex called the apoptosome – a scaffold consisting of cytochrome *c* bound to dATP and the cytochrome *c* apoptotic protease activating factor 1 (Apaf1). The apoptosome activates caspase-9 through its N-terminal caspase recruitment domain (CARD) and caspase-9 subsequently activates caspase-3 and caspase-7 (Crawford and Wells, 2011). To date, there is no evidence that such multiprotein pro-death complexes capable of activating PCD-related proteases exist in plants. However, the presence of a similar mechanism has been indirectly observed for plants. Whereas in animals, recombinant Bax protein is responsible for the release of cytochrome *c* from mitochondria, it also induces a response similar to a HR and a cell death response in tobacco (Lacomme and Santa Cruz, 1999). Additionally, when expressed in tobacco, the antiapoptotic protein Bcl-xL can confer resistance to death induced by UV-B irradiation and by paraquat (Mitsuhara et al., 1999), or by *Tobacco mosaic virus* protein p50 (Solovieva et al., 2013). However, Bcl-2 family orthologs are absent in plants, and this process which is similar to apoptotic cell death is achieved through other unidentified proteins.

## Transduction of a Signal: Which Proteolytic Cascades Exist in Plants?

Once a protease becomes active, it can change conformation and interact with other proteases (Figure 1B). As mentioned, the main executioners of apoptosis in animals are the caspases that act through the proteolytic cascades. Caspases can manage the two-step activation of PCD through the recruitment of initiator (caspases-2, -8, -9, -10) and effector (caspases-3, -6, -7) caspases (Crawford and Wells, 2011). How the initiator caspases cause the activation of effector caspases is through cleavages of a number of other proteases or proapoptotic substrates leading to death of the cell (Galluzzi et al., 2018). Apoptosis is characterized by YVADase, DEVDase, VEIDase and other activities (Kidd, 1998), which correspond to activities of caspases-1, -3, and -6, respectively.

Despite close homologs of caspases being absent in plants, proteases that belong to the same family of C14 cysteine proteases are present, called the metacaspases. Of interest is that metacaspases are lysine- and arginine-specific, unlike the aspartate-specific caspases, suggesting that metacaspases may not be directly responsible for similar caspase activities found in plants (Fagundes et al., 2015). However, type I metacaspases (AtMC1, AtMC2) are strongly associated with an autolytic type of PCD and plant immunity (Coll et al., 2010), whereas type II metacaspases from *Populus tremula* × *tremuloides* PttMC13 and PttMC14 are able to cleave PLCP, RD21 during xylem elements cell death (Bollhoner et al., 2018).

There are also many studies supporting the involvement of proteases other than metacaspases in plant PCD. Not only do cysteine proteases, such as VPEs, exhibit caspase-like activity (Hatsugai et al., 2004, 2009; Zhang et al., 2010), but proteasome subunit PBA1 (Hatsugai et al., 2009) and subtilases (Coffeen and Wolpert, 2004; Chichkova et al., 2010) were also shown to display same activity. Moreover, there are also proteases that do not exhibit caspase-like activity at all, but are closely associated with different types of PCD. For example, PLCPs are associated with pPCD [cathepsin B, RD21 (Gilroy et al., 2007; McLellan et al., 2009; Shindo et al., 2012)] and dPCD [CEP1, NtCP14, XCP1, XCP2 (Avci et al., 2008; Ruckh et al., 2012; Bollhoner et al., 2013; Salvesen et al., 2016)]. In addition, serine protease P69B is cleaved by apoplastic metalloproteases Sl2- and Sl3-MMPs (Li et al., 2015; Zimmermann et al., 2016) and regulates cell death in the tomato plant in response to *Botrytis cinerea* infection and *Pst*DC3000. Known examples of proteases that are involved in plant PCD are summarized in the Supplementary Table.

Based on the animal apoptotic pathway, the initiator-effector model was also proposed for the metacaspases (Rocha et al., 2017). Type I metacaspases undergo autocatalytic processing and can activate type II metacaspases. Due to the limited data, it is still difficult to assign the role of an initiator or effector protease for the “non-metacaspase” proteases that are involved in PCD. Moreover, there is a consensus, that cysteine proteases may not be universal regulators of PCD in plants as they are in animals (Sueldo and van der Hoorn, 2017) and may be they are unessential for plant PCD.

Recently, the question of whether proteolytic cascades exist in plants was addressed, and a specific requirement for two proteases to form a protease-substrate link was suggested (Paulus and van der Hoorn, 2019). It is certain, that although caspases are absent in plants, and caspase-like activity is not the only activity that characterizes plant PCD, it does lead to similar to animal apoptotic traits such as cytoplasm shrinkage, chromatin condensation, and nucleus fragmentation (Van Doorn et al., 2011). Based on this data, we believe that death-inducing cascades do exist in plants and their participants are different in origin, but similar in function. Recently, it was used for the identification of sites of hydrolysis by endogenous proteases during biotic stress (Balakireva et al., 2018). It was shown that during the early response of wheat to different pathogens, caspase-like and metacaspase-like activities are not required, while immune response is still triggered and, apparently, is held by some other proteases, which confirms our assumptions in a way.

## Execution of a Signal: Is PCD Derived From the Same Signaling Pathways in Both Animals and Plants?

It is true that plants and animals differ in a number of ways, firstly, due to photoautotrophic growth, absence of mobility and the presence of a semirigid cell wall. Independent evolution of animals and plants resulted in the development of analogous, but non-conserved protein structure and signaling pathways. One striking example is the animal Toll-like receptors. In plants, the equivalent is the receptor-like kinase (Ausubel, 2005). Both of them have a C-terminal leucine-rich repeat domain, and the cytoplasmic domains from the proteins are not conserved but are able to perform analogous functions. However, the downstream signaling events are very conserved among eukaryotic organisms such as the activation of mitogen-activated protease kinase (MAPK) cascades (Dong et al., 2002; Pitzschke et al., 2009).

Apoptosis itself is very conserved among metazoans and fungi (Crawford and Wells, 2011). Cleavage of a substrate by proteases at a specific site can result in two outcomes, the loss or gain of protein function (Figure 1C). In this manner, one effect of cleavage by caspases for a large number of their substrates was analyzed (Fischer et al., 2003) and made clear that the majority of substrates lose their function after hydrolytic cleavage which leads to a shutdown of almost all pathways essential for vital activity. However, some of the substrates become active after hydrolysis such as cytokines, protein kinases, and regulatory proteins essential for signal transduction.

It is very important to note that the analysis of caspase sites, their substrates and appropriate pathways in mice, *Drosophila* and *Caenorhabditis elegans*, which represent 600 million years of evolution, highlight that such sites are conserved over a relatively short evolutionary timeframe, in comparison to the lengthy timeframes of signaling pathways (Crawford et al., 2012). For example, the Tudor Staphylococcal Nuclease (TSN) protein is essential for the activation of transcription, mRNA splicing and RNA silencing, a pathway highly conserved among eukaryotes (Ausubel, 2005) in which TSN can be cleaved by both human caspase-3 and metacaspase mcII-Pa from Norway spruce (Sundstrom et al., 2009). Similarly, poly (ADP-ribose) polymerase (PARP) which is involved in the conserved pathway for DNA repair, is a substrate of human caspases and two metacaspases (MCA1 and MCA2) from the fungi *Podospora anserine* (Strobel and Osiewacz, 2013). Collectively, these findings indeed support the idea that eukaryotes share conserved signaling pathways that can be targeted by PCD proteases which are functionally similar but structurally unique (e.g., caspase-3 vs. metacaspase mcII-Pa, and others).

Additionally, another excellent example is a membrane receptor protein which can be cleaved by the tomato P69C protease and includes a leucine-rich repeat (Tornero et al., 1996). This protein can also be categorized into a group of membrane receptors that can also be targeted by caspases (Figure 1C). Finally, phosphoenolpyruvate carboxykinase 1 (PEPCK1) can be cleaved and activated by AtMC9 (Tsiatsiani et al., 2013). Other examples of known substrates of plant proteases are summarized in the Supplementary Table.

## Conclusion

Considering all the findings supporting the involvement of proteases in plant PCD, it is clear that this research area is relatively unexplored. To date, not a single proteolytic cascade in any plant has been linked to the PCD-related process. And the question posed is “why is the area of plan PCD so fragmentary?” Firstly, great emphasis has been placed on the study of human forms of PCD because of its immense therapeutic value and which serves as a good paradigm. Secondly, extrapolating such findings to the plant system has been slow due to a lack of methodology needed to yield findings in a timely manner. Thirdly, plant genomes contain many duplicated genes, especially in such organisms as hexaploid *Triticum aestivum*, which makes it difficult to perform knock-out studies.

To help matters, there has been some promising advances that have been recently introduced into plant science. “Big data” analytics are increasingly being used to discover hidden patterns, correlations and other insights from fragmented studies (Gharajeh, 2018). Additionally, genome-wide gene expression profiling and other “omics” technologies are indeed needed, started with proteomics approaches which are now becoming widely used for studying various aspects of plant death.

Plants do have their own features, their signaling networks do have a high level of functional redundancy. With similar parts of related pathways functionally compensating and substitutional for each other (Sewelam et al., 2016). Nevertheless, we believe that across eukaryotes its these pathways that are the most conserved rather than the regulatory proteins which constitute them. We assume that although proteases themselves (caspase vs. metacaspase) and their specificities (D-specific vs. R-, K-specific) are functionally giving rise to different morphologically diverse forms of PCD between animals and plants, such distinctions cannot be clearly made at this juncture in time.

## Data Availability

All datasets analyzed for this study are cited in the manuscript and the Supplementary Files.

## Author Contributions

AZ conceived an original idea for a review. AB wrote the manuscript and created a figure.

## Conflict of Interest Statement

The authors declare that the research was conducted in the absence of any commercial or financial relationships that could be construed as a potential conflict of interest.
