# Supplementary material for: Cutting Out the Gaps Between Proteases and Programmed Cell Death
Source: Front Plant Sci. 2019 Jun 4;10:704. doi: 10.3389/fpls.2019.00704 (PMC6558192; doi:10.3389/fpls.2019.00704)
Supplement: Supplementary file 1 [file Table_1.docx]

**Supplementary table.** Protease involvement in PCD in plants. Adapted from (Balakireva and Zamyatnin, 2018; Buono et al., 2019; Zamyatnin  Jr., 2015).

| Plant species | Plant protease | Family | Identified substrates | Function/Phenotype | Ref. |
| --- | --- | --- | --- | --- | --- |
| *Arabidopsis thaliana* L. | AtMC1, AtMC2 | Cys, C14B | ND | Suppression of hypersensitive cell death response upon infection with avirulent pathogen, AtMC1 and AtMC2 antagonistically control lsd1 runaway cell death | (Coll et al., 2010) |
|  | AtMC9 | Cys, C14B | GRI protein, PEPCK1, AtSerpin1 | Effector of PCD activation, Xylem cell death, degradation of vessel cell contents after vacuolar rupture | (Bollhoner et al., 2013, 2018) |
|  | CathB | Cys, C1A | ND | Required for the HR and disease resistance induced by non-host bacterial pathogens, positive regulatory role in senescence | (Gilroy et al., 2007; McLellan et al., 2009) |
|  | RD21 | Cys, C1A | ND | ‘Pro-death’ signal activated during elicitation of cell death, targeted by plant AtSerpin1, AtWSCP; processed by PttMC13 and PttMC14 | (Gu et al., 2012; Shindo et al., 2012) |
|  | RD19A | Cys, C1A | ND | RRS1-R-mediated resistance, inhibited by effector PopP2 | (Bernoux et al., 2008) |
|  | VPEs | Cys, C13 | Storage proteins (12S globulins and 2S albumins) | Activate vacuolar enzymes and disintegrate the vacuolar membrane to release hydrolytic enzymes during PCD, involved in the HR elicited by infection with TMV | (Hatsugai et al., 2004, 2015) |
|  | AtCEP1 | Cys, C1A | ND | Restriction of powdery mildew controlling late stages of compatible interaction including late epidermal PCD | (Howing et al., 2014) |
|  | PBA1 | Thr, T1B | ND | Caspase-3-like (DEVDase) activity in the vacuolar and plasma membranes proteasome-regulating membrane fusion | (Hatsugai et al., 2009) |
|  | AtSBT5.2(a and b) | Ser, S8 | ND | Independent from protease activity attenuation of MYB30-mediated HR | (Serrano et al., 2016) |
|  | SAG12 | Cys, C1A | ND | Its expression is induced during senescence and pathogen-induced cell death | (Singh et al., 2013)s |
|  | XCP1, XCP2 | Cys, C1A | ND | Post-mortem clearance | (Avci et al., 2008) |
|  | At2-MMP | Metallo, M10 | ND | Involvement in late flowering and early senescence | (Golldack et al., 2002) |
|  | FtSH4 | Metallo, M10 | ND | Involved in leaf senescence via regulation of WRKY-dependent salicylic acid accumulation and signaling | (Zhang et al., 2017) |
|  | AtCP51 | Cys, C1A | ND | CP51 critically mediates tapetum stability and pollen exine formation | (Yang et al., 2014) |
| *Picea abies* H. Karst. | mcII-Pa | Cys, C14 | Tudor Staphylococcal Nuclease (TSN) | Induces autophagy, which triggers RCD mechanisms during the terminal differentiation of embryonic suspensor cells, and participates in further development of RCD | (Bozhkov et al., 2005) |
| *Solanum lycopersicum* L*.* | P69B | Ser, S8 | ND | Local apoplast surveillance, substrate of Sl2-, Sl3-MMP, positive regulator of PCD | (Tian et al., 2005; Zimmermann et al., 2016) |
|  | P69C | Ser, S8 | Leucine-rich repeat protein | Leucine-rich repeat protein processing | (Tornero et al., 1996) |
|  | Sl2-, Sl3-MMPs | Metallo, M10A | P69B | Extracellular cascade of epidermal cell death | (Zimmermann et al., 2016) |
|  | CYP1 | Cys, C1A | ND | Involved in HR reactions | (Bar-Ziv et al., 2015) |
|  | C14 | Cys, C1A | ND | Defense-related secretion in haustoriated plant cells | (Bozkurt et al., 2011) |
|  | Sl-SBT3 | Ser, S8 | ND | Caspase-3-like DEVDase activity, HR-like PCD induction | (Cedzich et al., 2009)s |
| *Populus tremula x tremuloides* | PttMC13, PttMC14 | Cys, C14B | RD21, TSN, PASPA3 | Type II metacaspases, AtMC9 homologues, involvement of stress granules in the metacaspase-TSN pathway and xylem vessel and fiber cells PCD, processing of RD21, TSN, PASPA3—postmortem autolytic processes | (Bollhoner et al., 2018) |
| *Solanum tuberosum L.* | StSBTc-3 | Ser, S8 | ND | Caspase-3-like DEVDase activity, HR-like PCD induction | (Cedzich et al., 2009) |
| *Avena sativa* L. | Saspase | Ser, S8 | RuBisCO | RuBisCO proteolysis in victorin-induced PCD, IETDase and LEHDase activities | (Coffeen and Wolpert, 2004) |
| *Oryza sativa* L*.* | OsAP25, OsAP37 | Asp, A1 | ND | Promotion of tapetal cell death | (Niu et al., 2013) |
|  | UNDEAD | Asp, A1 | ND | Tapetal programmed cell death | (Phan et al., 2011) |
| *Nicotiana tabacum* L. | Phytaspase | Ser, S8 | VirD2 from *Agrobacterium tumefasciens* | Activated in tobacco mosaic virus (TMV)-induced HR, VirD2 cleavage preventing protein transport to nucleus, VEIDase, IETDase, LEHDase, and VDVADase | (Chichkova et al., 2004) |

Avci, U., Earl Petzold, H., Ismail, I. O., Beers, E. P., and Haigler, C. H. (2008). Cysteine proteases XCP1 and XCP2 aid micro-autolysis within the intact central vacuole during xylogenesis in Arabidopsis roots. *Plant J.* 56, 303–315. doi:10.1111/j.1365-313X.2008.03592.x.

Balakireva, A. V., and Zamyatnin, A. A. (2018). Indispensable role of proteases in plant innate immunity. *Int. J. Mol. Sci.* 19. doi:10.3390/ijms19020629.

Bar-Ziv, A., Levy, Y., Citovsky, V., and Gafni, Y. (2015). The Tomato yellow leaf curl virus (TYLCV) V2 protein inhibits enzymatic activity of the host papain-like cysteine protease CYP1. *Biochem Biophys Res Commun* 460, 525–529. doi:10.1016/j.bbrc.2015.03.063.

Bernoux, M., Timmers, T., Jauneau, A., Briere, C., de Wit, P. J., Marco, Y., et al. (2008). RD19, an Arabidopsis cysteine protease required for RRS1-R-mediated resistance, is relocalized to the nucleus by the Ralstonia solanacearum PopP2 effector. *Plant Cell* 20, 2252–2264. doi:10.1105/tpc.108.058685.

Bollhoner, B., Jokipii-Lukkari, S., Bygdell, J., Stael, S., Adriasola, M., Muniz, L., et al. (2018). The function of two type II metacaspases in woody tissues of Populus trees. *New Phytol* 217, 1551–1565. doi:10.1111/nph.14945.

Bollhoner, B., Zhang, B., Stael, S., Denance, N., Overmyer, K., Goffner, D., et al. (2013). Post mortem function of AtMC9 in xylem vessel elements. *New Phytol* 200, 498–510. doi:10.1111/nph.12387.

Bozhkov, P. V, Suarez, M. F., Filonova, L. H., Daniel, G., Zamyatnin Jr., A. A., Rodriguez-Nieto, S., et al. (2005). Cysteine protease mcII-Pa executes programmed cell death during plant embryogenesis. *Proc Natl Acad Sci U S A* 102, 14463–14468. doi:10.1073/pnas.0506948102.

Bozkurt, T. O., Schornack, S., Win, J., Shindo, T., Ilyas, M., Oliva, R., et al. (2011). Phytophthora infestans effector AVRblb2 prevents secretion of a plant immune protease at the haustorial interface. *Proc Natl Acad Sci U S A* 108, 20832–20837. doi:10.1073/pnas.1112708109.

Buono, R. A., Hudecek, R., and Nowack, M. K. (2019). Plant proteases during developmental programmed cell death. *J. Exp. Bot.* 70, 2097–2112. doi:10.1093/jxb/erz072.

Cedzich, A., Huttenlocher, F., Kuhn, B. M., Pfannstiel, J., Gabler, L., Stintzi, A., et al. (2009). The protease-associated domain and C-terminal extension are required for zymogen processing, sorting within the secretory pathway, and activity of tomato subtilase 3 (SlSBT3). *J Biol Chem* 284, 14068–14078. doi:10.1074/jbc.M900370200.

Chichkova, N. V, Kim, S. H., Titova, E. S., Kalkum, M., Morozov, V. S., Rubtsov, Y. P., et al. (2004). A plant caspase-like protease activated during the hypersensitive response. *Plant Cell* 16, 157–171. doi:10.1105/tpc.017889.

Coffeen, W. C., and Wolpert, T. J. (2004). Purification and characterization of serine proteases that exhibit caspase-like activity and are associated with programmed cell death in Avena sativa. *Plant Cell* 16, 857–873. doi:10.1105/tpc.017947.

Coll, N. S., Vercammen, D., Smidler, A., Clover, C., Van Breusegem, F., Dangl, J. L., et al. (2010). Arabidopsis type I metacaspases control cell death. *Science (80-. ).* 330, 1393–1397. doi:10.1126/science.1194980.

Gilroy, E. M., Hein, I., van der Hoorn, R., Boevink, P. C., Venter, E., McLellan, H., et al. (2007). Involvement of cathepsin B in the plant disease resistance hypersensitive response. *Plant J* 52, 1–13. doi:10.1111/j.1365-313X.2007.03226.x.

Golldack, D., Popova, O. V., and Dietz, K. J. (2002). Mutation of the matrix metalloproteinase At2-MMP inhibits growth and causes late flowering and early senescence in Arabidopsis. *J. Biol. Chem.* 277, 5541–5547. doi:10.1074/jbc.M106197200.

Gu, C., Shabab, M., Strasser, R., Wolters, P. J., Shindo, T., Niemer, M., et al. (2012). Post-translational regulation and trafficking of the granulin-containing protease rd21 of arabidopsis thaliana. *PLoS One* 7, 1–11. doi:10.1371/journal.pone.0032422.

Hatsugai, N., Iwasaki, S., Tamura, K., Kondo, M., Fuji, K., Ogasawara, K., et al. (2009). A novel membrane fusion-mediated plant immunity against bacterial pathogens. *Genes Dev* 23, 2496–2506. doi:10.1101/gad.1825209.

Hatsugai, N., Kuroyanagi, M., Yamada, K., Meshi, T., Tsuda, S., Kondo, M., et al. (2004). A plant vacuolar protease, VPE, mediates virus-induced hypersensitive cell death. *Science (80-. ).* 305, 855–858. doi:10.1126/science.1099859.

Hatsugai, N., Yamada, K., Goto-Yamada, S., and Hara-Nishimura, I. (2015). Vacuolar processing enzyme in plant programmed cell death. *Front. Plant Sci.* 6, 1–11. doi:10.3389/fpls.2015.00234.

Howing, T., Huesmann, C., Hoefle, C., Nagel, M. K., Isono, E., Huckelhoven, R., et al. (2014). Endoplasmic reticulum KDEL-tailed cysteine endopeptidase 1 of Arabidopsis (AtCEP1) is involved in pathogen defense. *Front Plant Sci* 5, 58. doi:10.3389/fpls.2014.00058.

McLellan, H., Gilroy, E. M., Yun, B. W., Birch, P. R., and Loake, G. J. (2009). Functional redundancy in the Arabidopsis Cathepsin B gene family contributes to basal defence, the hypersensitive response and senescence. *New Phytol* 183, 408–418. doi:10.1111/j.1469-8137.2009.02865.x.

Niu, N., Liang, W., Yang, X., Jin, W., Wilson, Z. A., Hu, J., et al. (2013). EAT1 promotes tapetal cell death by regulating aspartic proteases during male reproductive development in rice. *Nat Commun* 4, 1445. doi:10.1038/ncomms2396.

Phan, H. A., Iacuone, S., Li, S. F., and Parish, R. W. (2011). The MYB80 Transcription Factor Is Required for Pollen Development and the Regulation of Tapetal Programmed Cell Death in Arabidopsis thaliana . *Plant Cell* 23, 2209–2224. doi:10.1105/tpc.110.082651.

Serrano, I., Buscaill, P., Audran, C., Pouzet, C., Jauneau, A., and Rivas, S. (2016). A non canonical subtilase attenuates the transcriptional activation of defence responses in Arabidopsis thaliana. *Elife* 5. doi:10.7554/eLife.19755.

Shindo, T., Misas-Villamil, J. C., Horger, A. C., Song, J., and van der Hoorn, R. A. (2012). A role in immunity for Arabidopsis cysteine protease RD21, the ortholog of the tomato immune protease C14. *PLoS One* 7, e29317. doi:10.1371/journal.pone.0029317.

Singh, S., Giri, M. K., Singh, P. K., Siddiqui, A., and Nandi, A. K. (2013). Down-regulation of OsSAG12-1 results in enhanced senescence and pathogen-induced cell death in transgenic rice plants. *J. Biosci.* 38, 583–592. doi:10.1007/s12038-013-9334-7.

Tian, M., Benedetti, B., and Kamoun, S. (2005). A Second Kazal-like protease inhibitor from Phytophthora infestans inhibits and interacts with the apoplastic pathogenesis-related protease P69B of tomato. *Plant Physiol* 138, 1785–1793. doi:10.1104/pp.105.061226.

Tornero, P., Mayda, E., Gómez, M. D., Cañas, L., Conejero, V., and Vera, P. (1996). Characterization of LRP, a leucine-rich repeat (LRR) protein from tomato plants that is processed during pathogenesis. *Plant J.* 10, 315–330. doi:10.1046/j.1365-313X.1996.10020315.x.

Yang, Y., Dong, C., Yu, J., Shi, L., Tong, C., Li, Z., et al. (2014). Cysteine Protease 51 (CP51), an anther-specific cysteine protease gene, is essential for pollen exine formation in Arabidopsis. *Plant Cell. Tissue Organ Cult.* 119, 383–397. doi:10.1007/s11240-014-0542-0.

Zamyatnin Jr., A. A. (2015). Plant Proteases Involved in Regulated Cell Death. *Biochem.* 80, 1701–1715. doi:10.1134/S0006297915130064.

Zhang, S., Li, C., Wang, R., Chen, Y., Shu, S., Huang, R., et al. (2017). The Arabidopsis Mitochondrial Protease FtSH4 Is Involved in Leaf Senescence via Regulation of WRKY-Dependent Salicylic Acid Accumulation and Signaling. *Plant Physiol.* 173, 2294–2307. doi:10.1104/pp.16.00008.

Zimmermann, D., Gomez-Barrera, J. A., Pasule, C., Brack-Frick, U. B., Sieferer, E., Nicholson, T. M., et al. (2016). Cell Death Control by Matrix Metalloproteinases. *Plant Physiol* 171, 1456–1469. doi:10.1104/pp.16.00513.
